# Emotionally Biased Cognitive Processes: The Weakest Link Predicts Prospective Changes in Depressive Symptom Severity

**DOI:** 10.1371/journal.pone.0124457

**Published:** 2015-05-07

**Authors:** Jonas Everaert, Wouter Duyck, Ernst H. W. Koster

**Affiliations:** 1 Department of Experimental Clinical and Health Psychology, Ghent University, Ghent, Belgium; 2 Department of Experimental Psychology, Ghent University, Ghent, Belgium; University of California, San Francisco, UNITED STATES

## Abstract

Emotional biases in attention, interpretation, and memory are predictive of future depressive symptoms. It remains unknown, however, how these biased cognitive processes interact to predict depressive symptom levels in the long-term. In the present study, we tested the predictive value of two integrative approaches to model relations between multiple biased cognitive processes, namely the additive (i.e., cognitive processes have a cumulative effect) vs. the weakest link (i.e., the dominant pathogenic process is important) model. We also tested whether these integrative models interacted with perceived stress to predict prospective changes in depressive symptom severity. At Time 1, participants completed measures of depressive symptom severity and emotional biases in attention, interpretation, and memory. At Time 2, one year later, participants were reassessed to determine depressive symptom levels and perceived stress. Results revealed that the weakest link model had incremental validity over the additive model in predicting prospective changes in depressive symptoms, though both models explained a significant proportion of variance in the change in depressive symptoms from Time 1 to Time 2. None of the integrative models interacted with perceived stress to predict changes in depressive symptomatology. These findings suggest that the best cognitive marker of the evolution in depressive symptoms is the cognitive process that is dominantly biased toward negative material, which operates independent from experienced stress. This highlights the importance of considering idiographic cognitive profiles with multiple cognitive processes for understanding and modifying effects of cognitive biases in depression.

## Introduction

Depression is a prevalent and burdensome disorder [1] closely associated with emotional distortions in cognition: Individuals with elevated depressive symptom levels selectively attend to negative material [2], draw more negative interpretations on ambiguous information [3], and recall disproportionately more negative memories [4,5]. Clarifying how emotional biases in these basic cognitive processes are involved in the course of depressive symptoms over extended time remains a major challenge for contemporary clinical scientists. Indeed, emotional biases in attention [6], interpretation [7], and memory [8] are individually predictive of future depressive symptoms, but how these related, yet distinctive, aspects of cognition interact to predict prospective changes in depressive symptom severity remains unknown. An integrative perspective to surpass individual bias effects seems necessary to advance current knowledge on how emotionally biased cognitive processes contribute to depressive symptoms.

Integrative approaches to understand how cognitive factors work together in emotional disorders are relatively new [9,10]. Two important approaches have been proposed to conceptualize the longitudinal impact of multiple depression-linked distortions in cognition [11]. First, the additive approach assumes that the severity of distorted cognitive factors has a cumulative effect, such that the risk to develop depressive symptoms increases with each additional factor. Applied to emotionally biased cognitive processes, the model predicts that individuals with more severe negative biases in multiple processes are at greater risk to develop depressive symptoms than individuals with fewer negatively biased processes. Second, a weakest link approach predicts that the course of depressive symptoms depends on the most pathogenic cognitive factor and not on the number of factors. The best marker of future increases in depressive symptoms would then be the cognitive process that is dominantly biased toward negative material. Note that several cognitive science approaches to depression hypothesize that distorted cognitive processes elevate depression risk under high levels of stress [12]. This means that biased aspects of cognition and their combined effects predict changes in depressive symptoms through their interaction with perceived stress.

Research testing integrated models of distorted cognition as predictors of future depressive symptoms in adult samples is at the early stages. In research modeling effects of *content* aspects of cognition (e.g., questionnaire measures of dysfunctional attitudes and self-esteem) longitudinally, both the weakest link [13] and additive [14,15] model received support. However, studies contrasting these approaches have yielded mixed evidence for the model with the greatest predictive power. One study reported a high correlation (*r* = .93) between the weakest link and the additive model suggesting redundancy [16]. By contrast, another study observed greater power of the weakest link over the additive model in predicting prospective changes in depressive symptoms [17]. Data regarding integrated models × stress interactions are also mixed. While one study supports interactions between stress and integrative models [16], the other study found the interaction did not significantly predict additional sources of variance [17].

In research modeling longitudinal effects of *cognitive processes* (e.g., attention, memory), one study investigated whether prospective changes in depressive symptomatology and recovery status are predicted by multiple cognitive processes in a clinically depressed sample [18]. Neither attention nor memory bias was related to recovery at 9 months follow-up, and only memory for positive information at baseline was associated with lower symptom severity at follow-up. Although this study examined the predictive value of multiple biased cognitive processes individually, neither integrative models nor stress-interactions were tested. Unfortunately, this type of research on emotional biases in basic cognitive processes is currently lacking. Clarifying the relation between biased cognitive processes and depressive symptoms seems instrumental in understanding both depression and aspects of cognition.

The present longitudinal study aimed to advance understanding of emotionally biased cognitive processes as predictors of changes in depressive symptoms by adopting an integrative perspective. A first aim was to apply the additive and weakest link approaches to depression-linked biases in attention, interpretation, and memory to contrast the models, testing their incremental utility. A second aim was to test whether integrative models interacted with perceived stress to predict the evolution in depressive symptoms.

## Methods

A one-year follow-up was conducted building on data of an earlier study [19]. This earlier study found that depression-linked biases in selection of attention and sustained attention regulate memory via different mechanisms: Attentional selection was associated with emotional memory via its relation with interpretation, while sustained attention was directly related to memory bias. The present study focuses on the predictive value of these biased cognitive processes for depression measures, one year later.

### Participants

All 71 undergraduate students (62 women) who participated in the cross-sectional study were invited to contribute to the Time 2 assessment. Fifty-three participants (49 women) completed both time assessments (74.65%). Participants were native Dutch speakers between 17 and 33 years with normal or corrected-to-normal vision. All individuals provided written informed consent and received 5 euro. The study was approved by the institutional review board at Ghent University.

### Time 1 assessment

In a 70-minute session, participants started with a scrambled sentences test to measure interpretation bias. A computerized version of the test presented 60 emotional (e.g., “am winner born loser a I”) and 40 neutral (e.g., “the I theatre visit cinema often”) scrambled sentences in fixed random order. This was to ensure that no more than two emotional scrambled sentences were presented consecutively within a block (to reduce priming effects) and the themes tapped into by the emotional scrambled sentences (e.g., self-esteem, future expectancies) were matched between blocks. There were 10 blocks, each comprising 6 emotional and 4 neutral sentences. Each sentence prompted participants to unscramble the item to form grammatically correct and meaningful statements using five of the six words (e.g., “I often visit the theatre”). Unscrambled solutions were registered via coded report. To reduce social desirable responses, participants memorized a six digit number (i.e., a cognitive load) before each block and were prompted to recall the number after the block. While a scrambled sentence was on-screen, participants’ eye movements were recorded to assess attention biases toward emotional target words (e.g., “winner” and “loser” in “am winner born loser a I”). Target words were matched on word length, word class, and word frequency. Emotional scrambled sentences—which could be solved in either a positive or a negative manner—were of interest to infer emotional biases in interpretation [7] and attention [20]. The ratio of negative over the total emotional unscrambled sentences served as an index of interpretation bias. Attentional selection bias was indexed by the total number of fixations on negative target words divided by the total number of fixations on positive and negative target words of the scrambled sentences. Analogous calculations on the fixation durations on positive and negative target words provided an index of sustained attention bias.

After the task, participants received 5 minutes to recall the constructed unscrambled sentences. Memory bias was computed by dividing the number of negatively unscrambled sentences recalled accurately by the total number of unscrambled emotional sentences recalled correctly. Finally, participants filled out the Beck Depression Inventory – II (BDI-II) [21,22] to assess depressive symptom severity. This questionnaire presents 21 statements to be rated on a scale from 0 to 3. The BDI-II has good reliability and validity in nonclinical and depressed samples [21,22]. The internal consistency was α = .92.

### Time 2 assessment

On average 368 days (*SD* = 25.66; range: 347–523 days) later, participants were reassessed to determine depressive symptoms and stress experienced prior to follow-up. Depressive symptoms were again measured by the BDI-II (internal consistency: α = .89). To control for stress levels experienced prior to follow-up, the Perceived Stress Scale (PSS) [23] assessed the degree to which participants appraised their life as stressful in the month prior to follow-up. The 10-item questionnaire presents disorder-unspecific items to provide a general estimation of how unpredictable, uncontrollable, and overloaded individuals have experienced their lives. Each item is rated on a scale from 0 (never) to 4 (very often). Research has supported the psychometric properties of the scale [24]. The internal consistency was α = .88 in this study.

### Bias composites

Bias indexes of attentional selection, sustained attention, interpretation, and memory were on the same metric, namely percentages reflecting preferential processing of negative over positive material [19]. An additive composite was computed by summing all four bias indexes per participant. A weakest link composite was computed by selecting the highest score of the bias indexes per participant. Each bias contributed to the weakest link composite. The dominant bias was in 26.42% of the participants attentional selection, in 26.42% sustained attention, in 18.87% interpretation, and in 28.30% memory bias.

## Results

### Descriptive statistics and attrition analysis

A BDI-II mean of 13.56 (*SD* = 9.57) was observed at Time 1 in the full sample. Attrition analyses indicated that participants who completed the Time 2 assessment (n = 53) reported lower BDI-II scores at Time 1, *M* = 11.70 (*SD* = 8.67), than participants who did not complete the Time 2 assessment (n = 18), *M* = 19.06 (*SD* = 10.22), *F*(1,69) = 8.83, *p*<.01. However, a broad range of BDI-II scores at Time 1 in the sample of completers was preserved: 34 individuals reported minimal, 6 mild, 11 moderate, and 2 severe depressive symptoms. Importantly, no differences emerged between completers and non-completers on the bias indexes (all *F*’s<1). At Time 2, a mean BDI-II score of 10.51 (*SD* = 8.02) and PSS score of 17.31 (*SD* = 6.92; range: 6–34) was observed. The BDI-II scores at Time 2 demonstrated a broad range: 32 individuals reported minimal, 13 mild, 7 moderate, and 1 severe symptom levels.

### Correlational analysis

The additive and weakest link composites were correlated and related to depressive symptom severity levels at both time assessments. Experienced stress was not related to the either composite score. Table 1 presents correlations between depressive symptoms, perceived stress, and cognitive bias composites.

**Table 1:** Correlations between depressive symptom severity, perceived stress and cognitive bias composites.

Variable	T1 BDI-II	T2 BDI-II	PSS	Weakest link
T1 BDI-II	—			
T2 BDI-II	.62^d^	—		
PSS	.57^d^	.78^d^	—	
Weakest link	.33^c^	.41^c^	.11	—
Additive	.52^d^	.46^c^	.26^a^	.75^d^

*Note1*.

^a^ *p*<.10

^b^ *p*<.05

^c^ *p*<.01

^d^ *p*<.001

*Note2*. BDI-II = Beck Depression Inventory—II; PSS = Perceived Stress Scale (measured at time 2). *Note3*. Weak to moderate correlations were observed between individual cognitive biases at Time 1. Sustained attention correlated with selective orienting, *r* = .54, *p*<.001, and memory, *r* = .39, *p*<.01, but not with interpretation, *r* = .18, *p* = .20. Selective orienting correlated with interpretation, *r* = .34, *p*<.05, but not with memory bias, *r* = .21, *p* = .13. Interpretation correlated with memory bias, *r* = .52, *p*<.001. None of the four cognitive biases were redundant and the strength of the observed correlations is similar to correlations reported between cognitive content variables [17].

### Prediction of depression symptoms at Time 2

To test the predictive value of each integrative model and its interaction with perceived stress, three-step hierarchical regression analyses were conducted per bias composite. In a first step, BDI-II scores at time 1 (T1 BDI-II) and PSS scores were entered to create a residual change score for BDI-II scores at time 2 (T2 BDI-II) and to control for proximal stress levels. The composite was added in a second step and its interaction with perceived stress in a third step. All predictors were z-transformed. Note that, to obtain composite-by-stress interaction indexes, the lowest value of the standardized scores was added to the variable before multiplying both variables [9]. For each analysis, collinearity statistics were within acceptable limits indicating low levels of multicollinearity (VIF’s<1.79, Tolerance’s>.55). Table 2 presents coefficients and statistics per tested model.

**Table 2:** Hierarchical regression models testing integrative models.

		Additive model				Weakest link model			
Predictor		*b*	*SE*_*b*_	*β*	*t*	*b*	*SE*_*b*_	*β*	*t*
Step 1	Constant	10.83	0.69		15.61^d^	10.83	0.69		15.61^d^
	T1 BDI-II	2.25	0.90	.26	2.50^b^	2.25	0.90	.26	2.50^b^
	PSS	5.11	0.82	.64	6.24^d^	5.11	0.82	.64	6.24^d^
Step 2	Constant	7.87	1.54		5.12^d^	8.00	0.92		8.67^d^
	T1 BDI-II	1.39	0.96	.16	1.45	1.34	0.82	.15	1.64
	PSS	5.14	0.79	.64	6.51^d^	5.30	0.72	.66	7.41^d^
	Composite	1.67	0.78	.20	2.13^b^	2.74	0.68	.31	4.06^d^
Step 3	Constant	7.66	1.54		4.98^d^	8.00	0.93		8.57^d^
	T1 BDI-II	1.31	0.95	.15	1.37	1.34	0.82	.15	1.62
	PSS	3.87	1.28	.48	3.02^c^	5.28	0.96	.66	5.49^d^
	Composite	0.52	1.20	.06	0.44	2.72	1.15	.31	2.36^b^
	Composite × PSS	0.71	0.56	.26	1.26	0.02	0.55	.00	0.03

*Note*.

^a^ *p*<.10

^b^ *p*≤.05

^c^ *p*<.01

^d^ *p*<.001

*Note2*. BDI-II = Beck Depression Inventory—II; PSS = Perceived Stress Scale.

Tests of the *additive model* revealed that T1 BDI-II and PSS accounted for 65.4% of the variance in step 1, *F*(2,49) = 46.22, *p*< .001. The additive composite in step 2 explained an additional 3.0% of the variance, *ΔF*(1,48) = 4.54, *p*<.05, and its interaction with stress in step 3 did not significantly add to the model, *ΔR* ^*2*^ = 1.0%, *ΔF*(1,47) = 1.59, *p* = .21. The variables included in step 2 accounted for 68.4% of the variance in depressive symptoms at Time 2, with PSS (*β* = .64, *p*<.001), the additive composite (*β* = .20, *p*<.05), but not T1 BDI-II (*β* = .16, *p* = .15) as significant predictors of T2 BDI-II.

Analysis of the *weakest link model* showed that adding T1 BDI-II and PSS in step 1 contributed significantly to the regression model, *F*(2,49) = 46.22, *p*<.001, *R* ^*2*^ = 65.4%. Introducing the weakest link composite in step 2 explained an additional 8.8%, *ΔF*(1,48) = 16.46, *p*<.001. Adding the weakest link × stress interaction in step 3 explained no additional variance, *ΔR* ^*2*^ = 0%, *ΔF*<1, *p* = .98. Of the variables included in step 2, not T1 BDI-II (*β* = .15, *p* = .11), but PSS (*β* = .66, *p*<.001) and the weakest link composite (*β* = .31, *p*<.001) predicted T2 BDI-II. Together, these variables accounted for 74.2% of the variation in depressive symptomatology at Time 2.

### Incremental utility

Prior analyses revealed that both integrative models predict the prospective change in depressive symptoms, with the weakest link model explaining a larger proportion of variance (8.8%) compared to the additive model (3.0%). To test the incremental utility of the weakest link model, a hierarchical regression analysis was conducted with T1 BDI-II and PSS added in step 1, and the additive and weakest link composites entered in step 2 and 3, respectively. Collinearity statistics were within acceptable limits (VIF<2.7, Tolerance>.44). Results showed that adding the weakest link composite in step 3 significantly added to the model including T1 BDI-II, PSS, and the additive composite, *ΔR* ^*2*^ = 6.2%, *ΔF*(1,47) = 11.39, *p*<.01. Of the variables in step 3, PSS (*β* = .66, *p*<.001) and the weakest link composite (*β* = .37, *p*<.01), but neither T1 BDI-II (*β* = .18, *p* = .08) nor the additive composite (*β* = -.09, *p* = .44) predicted T2 BDI-II.

### Potential confounds

Partial correlations controlling for T1 BDI-II showed that neither the number of days between Time 1 and Time 2 assessment, *r*_partial_ = .10, *p* = .50, nor gender, *r*_partial_ = .18, *p* = .20, nor age, *r*_partial_ = -.02, *p* = .89,were related to T2 BDI-II. The above findings are thus not confounded by these variables.

## Discussion

The present study is, to our knowledge, the first to examine emotionally biased cognitive processes in interaction with perceived stress as predictors of prospective changes in depressive symptomatology by adopting an integrative perspective. It was found that the weakest link model had incremental utility over the additive model in predicting the change in depressive symptoms, although both models significantly predicted the change in depressive symptoms over time. In addition to the large proportion of variance accounted by depressive symptoms at Time 1 and perceived stress levels (65.4%), the weakest link composite (8.8%) explained more than two times the variability in the evolution of symptomatology at Time 2 than the additive composite (3.2%). This suggests that the best cognitive marker of prospective fluctuations in depressive symptoms does not depend on the number of emotionally biased cognitive processes, but instead on the severity of emotional bias (toward negative material) in the most affected cognitive process. Notwithstanding the variance explained by perceived stress, stress did not interact with integrative models to predict changes in depressive symptoms. In line with prior studies [7,18], this finding suggests that cognitive biases can operate regardless of experienced stress, which contradicts predictions by several cognitive science approaches to depression [12]. However, cautious conclusions are warranted given that only few prospective studies have examined cognitive process × stress interactions and these have yielded inconsistent findings [16,17]. Note that the measure of stress in this study may have lacked sensitivity to detect idiographic changes. Despite the considerable variability in perceived stress scores to demonstrate interaction-effects, the baseline to rate stress may have differed across participants providing an inaccurate estimation of increases in stress prior to Time 2 assessment.

The present findings make two important contributions. First, they extend prior research in that this study applies integrative approaches to model longitudinal relations between multiple cognitive factors and depressive symptoms to the study of emotional biases in basic cognitive processes, which have generally been investigated in isolation and without stress-interactions [18]. The present observations add to research on cognitive content factors supporting the incremental validity of the weakest link over the additive approach [17], and diverge from research suggesting redundancy between the integrative models tested [16]. In line with the combined cognitive biases hypothesis stating that “*combinations of biases have a greater impact on disorders than if individual cognitive processes acted in isolation*” (p.224; [25]], the current data suggests that multiple *interactive* cognitive biases need to be considered to identify –within individuals– the bias with the greatest impact. Even though the best predictive potential may reside in one particular cognitive bias at one point in time, this biased cognitive process can still exert a strong influence other processing biases later on [19,26].

Second, the findings have practical implications for cognitive training methods that manipulate emotional biases in cognitive processes and have been implemented as tools to prevent future depressive symptoms [27]. If an individual’s weakest link is the best maker of future increases in depressive symptoms, cognitive training may be more effectively implemented after mapping an individual’s cognitive profile with various distorted aspects of cognition such that training can be tailored toward the cognitive process that is most affected in order to prevent future depressive symptoms. Note, that this study is the first to test the predictive value of integrative approaches to cognitive biases. Replication in clinical samples is required before such a strategy could be employed in clinical settings.

Several limitations of the present study should be acknowledged. A first limitation is that we did not examine whether the predictive power of multiple cognitive processes differs in first-onset versus recurrent depression. Future work will need to take into account the number of past depressive episodes to clarify differential predictive effects of integrative approaches. Another limitation is that we conducted the study in a sample with subclinical symptoms of depression and we did not take a diagnostic assessment of clinical depression. Instead, we investigated the predictive value of multiple cognitive processes in relation to prospective changes in self-reported symptoms. However, the present results remain of interest given that individuals with subclinical symptom levels experience significant symptomatic suffering, impaired role functioning, are at greater risk to develop clinical depression [28], and the cognitive biases under study here may contribute to this pathogenesis. Future research may extend the current work by examining the potency of integrated models to predict clinical outcomes such as recovery status in remitted and clinical samples. A third limitation is the absence of a measure reflecting depression-related deficits in cognitive control. Cognitive control processes may be an overarching mechanism operating across emotional biases in attention, interpretation, and memory [29]. When further investigating predictive effects of integrative models, it would be interesting to integrate cognitive control deficits into additive and weakest link models. A final limitation concerns the scale to evaluate perceived stress which may have not been sufficiently sensible to detect the stressful events experienced during the follow-up period. The scale measured perceived stress one month prior to follow-up to statistically control for proximal stress levels to examine the predictive value of the proposed integrative approaches. However, interview-based assessments that monitor the different stressful situations experienced during the full follow-up period may be preferred to test predictions by cognitive models of depression.

## Conclusion

This study investigated integrative effects of multiple emotional biases in basic cognitive processes as predictors of the future evolution of depressive symptoms. The weakest link model integrating attention, interpretation, and memory biases had incremental utility over the additive model, but did not interact with stress to predict the change in depressive symptomatology. This highlights the importance of considering multiple biased cognitive processes in evaluating effects of cognitive factors on the longitudinal course of depressive symptoms.
